# Leiomyosarcoma of Bone Arising in Association with a Bone Infarct

**DOI:** 10.1080/13577140220127558

**Published:** 2002-03

**Authors:** M. Petra, C. L. M. H. Gibbons, N. A. Athanasou

**Affiliations:** Department of Pathology Nuffield Department of Orthopaedic Surgery University of Oxford Nuffield Orthopaedic Centre Oxford OX3 7LD UK

## Abstract

Both primary leiomyosarcoma of bone and sarcoma arising in association with a bone infarct are rare events. In this case report we describe for the first time a case of leiomyosarcoma arising in a bone infarct. The tumour arose in a medullary infarct in the proximal femur of an elderly patient. As in other cases of sarcoma arising in a bone infarct, the prognosis was poor, the patient dying within 6 months of diagnosis.


*Sarcoma* (2002) **6**, 47–50

Taylor&Francis healthsciences

**CASE REPORT**

### Leiomyosarcoma of bone arising in association with a bone infarct

M. PETRA, C.L.M.H. GIBBONS & N.A. ATHANASOU

*Department of Pathology, Nuffield Department of Orthopaedic Surgery, University of Oxford, Nuffield Orthopaedic Centre, Oxford OX3 7LD, UK*

**Abstract**
Both primary leiomyosarcoma of bone and sarcoma arising in association with a bone infarct are rare events. In this case report we describe for the first time a case of leiomyosarcoma arising in a bone infarct. The tumour arose in a medullary infarct in the proximal femur of an elderly patient. As in other cases of sarcoma arising in a bone infarct, the prognosis was poor, the patient dying within 6 months of diagnosis.

**Key words:** *leiomyosarcoma, bone infarct, sarcoma*

### Introduction

Bone sarcoma arising secondary to infarction is a rare but well-documented entity.[1–3] Whether there is a causal association between bone infarction and sarcoma formation is not certain. It has been suggested that sarcomas arise by malignant transformation of reparative tissue within the infarcted bone. Sarcoma formation has been reported in association with both primary (idiopathic) bone infarcts as well as bone infarcts occurring secondary to a known underlying cause.

Leiomyosarcoma is a malignant smooth muscle tumour which very rarely occurs as a primary tumour of bone.[4–6] In this report, we document the first case of a leiomyosarcoma of bone arising in association with a medullary bone infarct.

### Case report

A 73-year-old man was admitted as an emergency after sustaining a subtrochanteric pathological fracture of his right femur; there was no previous history of limp or leg pain. Plain radiographs revealed a large mixed lytic and sclerotic lesion in the subtrochanteric region with loss of the medial cortex and permeative, ill-defined margins proximally (Fig. 1). The fracture was stabilised using a dynamic hip compression screw and plate. At the time of surgery, the fracture was noted to be associated with a large soft tissue mass, and bone biopsy at that time suggested a high-grade malignant spindle cell tumour, most likely a primary bone sarcoma. Further staging and screening investigations for metastatic disease including chest X-ray and CT, abdominal ultrasound, bone scan, blood biochemistry/haematology and estimation of serum prostate specific antigen levels were negative. The patient was referred to the NOC, Oxford, and underwent wide resection of the right proximal femur and reconstruction using a custom made hip endoprosthesis with a bipolar head (Stanmore, UK). Although clear margins of the removed bone segment were noted histologically, some concern was raised about microscopic margins in the soft tissue component and the patient had radiotherapy postoperatively. Histological findings were in keeping with those of primary leiomyosarcoma of bone. The patient died 5 months later.

### Radiological findings

Plain X-rays and magnetic resonance imaging (MRI) showed a large destructive osteolytic lesion in the proximal femur. The lytic area was permeative, poorly defined and associated with an area of increased density on plain X-rays (Fig. 1). In addition to the bone tumour, MRI of the right leg showed a soft tissue mass measuring 9 × 9 × 6 cm in diameter adjacent to the proximal femur on its medial and anterior aspects, the tumour displaced but did not invade the superficial femoral vessels; there were no skip lesions. The radiological differential diagnosis included metastatic carcinoma, lymphoma and primary bone sarcoma.

Correspondence to: N.A. Athanasou. Tel.: +44–1865–227619; Fax: +44–1865–745348; E-mail: nick.athanasou@noc.anglox.nhs.uk

1357–714X print/1369–1643 online/02/010047–04 © 2002 Taylor & Francis Ltd
DOI: 10.1080/13577140220127558

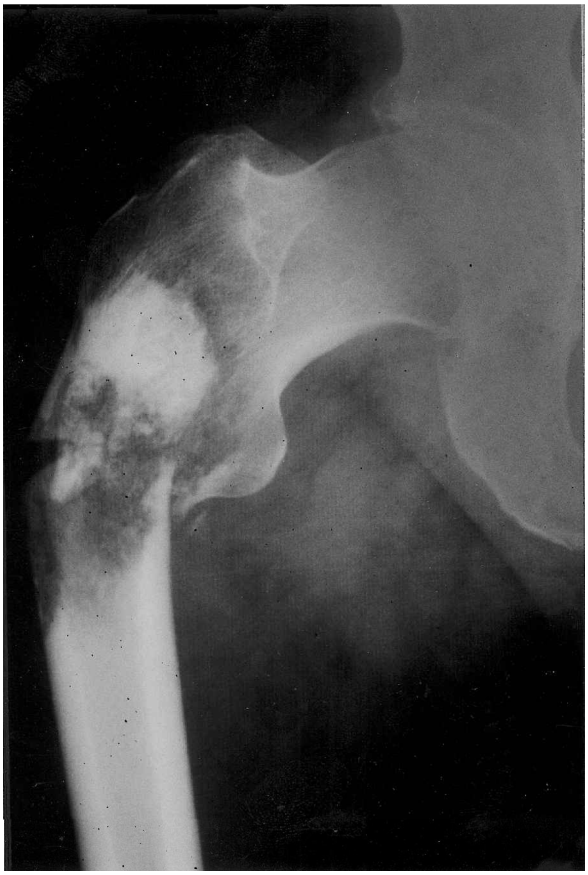

**Fig. 1.** *Preoperative radiograph of the proximal femur showing a subtrochanteric pathological fracture and a large, poorly defined lytic and sclerotic lesion.*

### Pathological findings

Grossly, the tumour was large and composed of firm pale tissue containing areas of haemorrhage and necrosis. An area of bone sclerosis was noted within the tumour which was present beneath the plate on the surface of the bone as well as within the track of the dynamic hip compression screws. The tumour measured 11 × 10 × 7 cm in maximum dimension and extended into surrounding tissue anteriorly and medially (Fig. 2).

Histological examination showed that the tumour was highly cellular and composed mainly of spindle-shaped cells arranged in fascicles (Fig. 3a). The spindle-shaped tumour cells had elongated vesicular, blunt-ended nuclei with prominent nucleoli; the cytoplasm was eosinophilic and contained small, often paranuclear vacuoles. Mitotic activity was brisk (up to five per ten high power fields) (Fig. 3b). The degree of cellularity, nuclear pleomorphism and amount of necrosis was variable in different parts of the tumour. In addition to a fascicular pattern of growth, a storiform pattern was also seen focally in parts of the tumour. Immunohistochemistry showed that the tumour cells were positive for vimentin, muscle-specific actin, smooth muscle actin and desmin (Fig. 3c). There was no staining for cytokeratin, S100 protein or epithelial

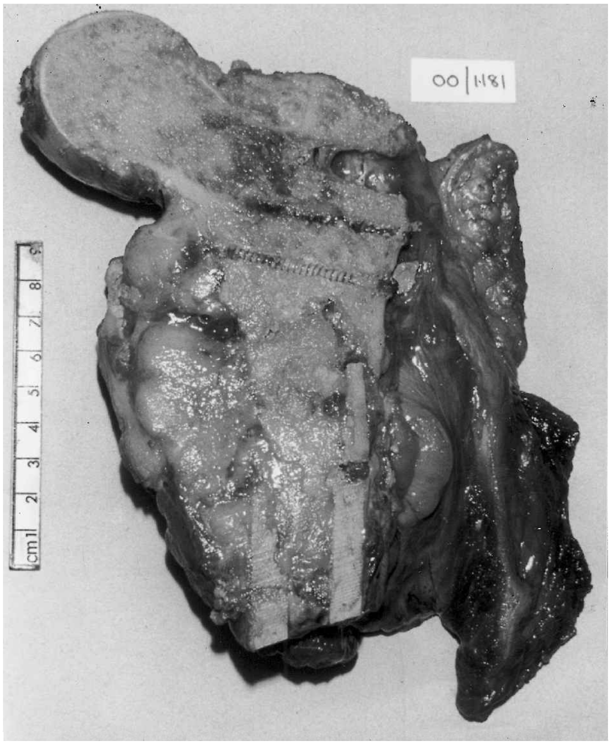

**Fig. 2.** *Gross appearance of the tumour in the proximal femur. The tumour extends along the screw tracts and has infiltrated through the medial cortex into the surrounding soft tissues.*

membrane antigen. Tumour cells were also positive for proliferating cell nuclear antigen. The tumour infiltrated medullary bone and extended through the cortex into surrounding soft tissues. Adjacent to the tumour, there was a distinct area of medullary bone infarction, consisting of sclerotic necrotic bone showing loss of osteocyte nuclei from lacunae (Fig. 3d). On the basis of morphological and immunohistochemical findings, the tumour was diagnosed as a primary leiomyosarcoma of bone arising in association with a medullary bone infarct; this diagnosis was confirmed by the UK National Bone Tumour Panel.

### Discussion

Although leiomyosarcoma is an extremely rare primary tumour of bone, over 60 cases have been described in the literature[1,2,4,8,9,11–15] To our knowledge, this is the first report of a leiomyosarcoma arising in association with a bone infarct.

Primary leiomyosarcomas of bone have been reported to occur over a wide age range. The main differential diagnosis is metastatic leiomyosarcoma, the primary tumour usually being uterine or gastrointestinal tract in origin. It has been suggested that primary osseous leiomyosarcomas arise from vascular smooth muscle cells within bone. Differentiation between a primary bone leiomyosarcoma and a leiomyosarcoma arising in soft tissue around bone may also prove difficult, particularly where the tumour

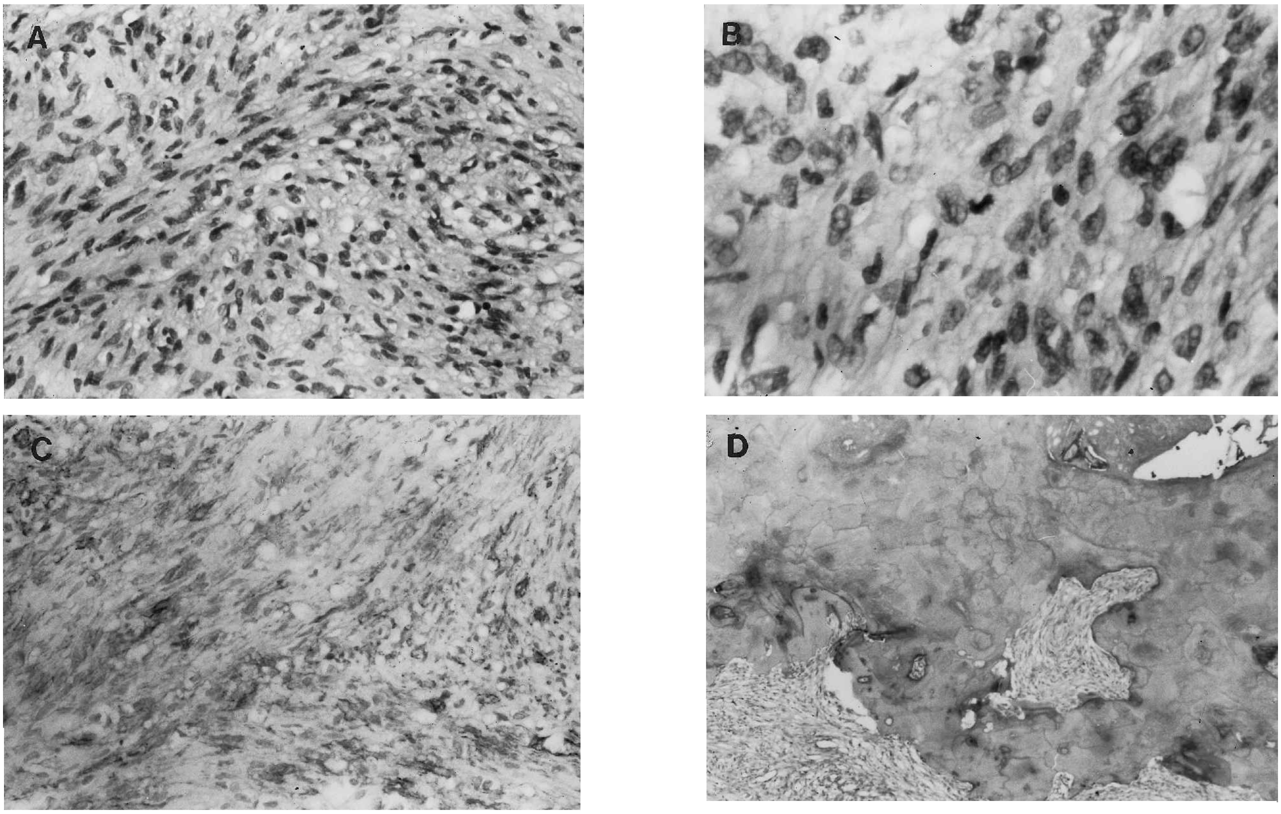

**Fig. 3.** *Photomicrograph of the tumour in the proximal femur showing the morphological features of the tumour under (a) low-power and (b) high-power. The tumour has a fascicular pattern and is composed of spindle-shaped cells showing brisk mitotic activity. In keeping with the diagnosis of leiomyosarcoma, the tumour cells express smooth muscle actin (c). The tumour arose in association with a sclerotic medullary bone infarct (d). (a,b,d) Haematoxylin and eosin, original magnification, × 200, × 400, × 600, respectively; (c) immunoperoxidase, original magnification, × 300.*

involves bones of the nasal or oral cavity. Primary leiomyosarcoma has been reported in long tubular bones of the extremities, bones of the axial skeleton and craniofacial bones, but has been noted most commonly in the femur. The fact that most leiomyosarcomas have been reported in the distal femur, where medullary infarcts are not uncommonly found, is worthy of note. Leiomyosarcoma occurring secondary to radiation therapy has also been described; this is of interest as radiation therapy can cause bone infarction.

Radiologically, leiomyosarcomas of bone are poorly defined and mainly osteolytic with a permeative or moth-eaten pattern of bone destruction; sclerotic areas are not usually found within primary or metastatic leiomyosarcomas involving bone. MRI and bone scans are used to stage the lesion and other radiological investigations are useful in excluding the possibility of metastatic disease. The histological differential diagnosis of leiomyosarcoma includes other malignant spindle cell tumours of bone such as fibrosarcoma, malignant fibrous histiocytoma and metastatic spindle cell carcinoma. The diagnosis of leiomyosarcoma is established (as in the present case) by immunohistochemical demonstration of a smooth muscle actin and other muscle markers on tumour cells. Electron microscopy can also be used to demonstrate ultrastructural evidence of smooth muscle differentiation of tumour cells.

Bone infarction is usually post-traumatic or idiopathic in nature, but can occur secondary to a variety of other conditions including decompression sickness and sickle cell anaemia. Sarcoma arising at the site of a bone infarct is a well-described entity.[1–3,16–19] The most common type of sarcoma associated with bone infarction is a malignant fibrous histiocytoma. Osteosarcoma, fibrosarcoma, angiosarcoma and malignant epithelioid haemangioendothelioma have also been reported. The incidence of sarcoma development in association with a bone infarct is low (0.6–1%). Most cases arise in adults, generally older than 40 years, and there is a slight male predominance. This complication has been reported to occur disproportionately in black patients (36% of cases in the review by Torres and Kyriakos[16]). Pain is the most common presenting symptom. Sarcomas have arisen in bone infarcts occurring secondary to radiation treatment and in this regard it should be noted that leiomyosarcoma of bone has been reported to have arisen following radiation treatment for a squamous carcinoma and mediastinal seminoma, a long latency period between treatment and sarcoma development being noted in both cases.[10]

It has been suggested that the pathogenesis of sarcomas arising in association with a bone infarct is the continuing reparative process, including vascular proliferation, that occurs around an area of osteonecrosis. Sarcomas are also known to arise in other lesions in which bone necrosis and continuing reparative changes are noted, including chronic osteomyelitis, laparotomy scars and radiation tissue damage. Bullough *et al.*,[20] in their study of 19 cases, noted that an area of bone infarction is frequently not replaced by normal tissue and that it is often surrounded by a rim of collagenous connective tissue. It has been estimated that in decompression workers the time interval between the event of bone infarction and sarcoma development is between 8 and 24 years. As in the present case, where there was no known predisposing cause for bone infarction, most cases of infarct-associated sarcoma have arisen in idiopathic medullary bone infarct.

In general, survival following sarcoma development in association with a bone infarct is poor.[1–3] Primary leiomyosarcoma of bone itself is known to behave aggressively and is associated with the poor prognosis, so it is not surprising that survival was not prolonged in our case. The surgical treatment of leiomyosarcoma of bone is similar to that of other primary malignant bone tumours. The role of adjuvant treatment and its effect on long-term prognosis needs to be evaluated.

### Acknowledgements

The authors wish to thank Caroline Costar for typing the manuscript.

### References

1. Furey JG, Ferrer-Torells M, Reagan JW. Fibrosarcoma arising at the site of bone infarcts. A report of two cases. *J Bone Joint Surg* 1960; 42A: 802–10.
2. Mirra JM, Bullough PG, Jacobs B, Huvos AG. Malignant fibrous histiocytoma and osteosarcoma in association with bone infarcts. Report of four cases, two in caisson workers. *J Bone Joint Surg* 1974; 56A: 932–40.
3. Desai P, Perino G, Present D, Steiner G. Sarcoma in association with bone infarcts. *Arch Pathol Lab Med* 1996; 120: 482–9.
4. Evans DMD, Sanerkin NG. Primary leiomyosarcoma of bone. *J Pathol* 1965; 90: 348–50.
5. Berlin O, Angervall K, Kindblom IG, Berlin IC, Stener B. Primary leiomyosarcoma of bone: a clinical, radiographic, pathologic-anatomic, and prognostic study of 16 cases. *Skeletal Radiol* 1987; 16: 364–76.
6. Eady JL, McKinney JD, McDonald EC. Primary leiomyosarcoma of bone. *J Bone Joint Surg* 1987; 69: 287–9.
7. Jundt G, Moll C, Nidecker A, Schilt R, Remagen W. Primary leiomyosarcoma of bone. *Hum Pathol* 1994; 25: 1205–12.
8. Khoddami M, Bedard YC, Bell RS, Kandel RA. Primary leiomyosarcoma of bone. Report of seven cases and review of the literature. *Arch Pathol Lab Med* 1996; 120: 671–5.
9. Meyers JL, Arocho J, Bernreuter W, Dunham W, Mazur MT. Leiomyosarcoma of bone: a cliniocopathologic, immunohistochemical, and ultrastructural study of five cases. *Cancer* 1991; 67: 1051–6.
10. Aoki T, Ozeki Y, Watanabe M, Tanaka S, Isaki H, Terahata S. Development of primary leiomyosarcoma of the sternum postirradiation: report of a case. *Jpn J Surg* 1998; 28: 1326–8.
11. Overgaard JM, Frederiksen P, Helming O, Jenson OM. Primary leiomyosarcoma of bone. *Cancer* 1977; 39: 1664–71.
12. Shamsuddin AK, Reyes F, Harvey JW, Toker C. Primary leiomyosarcoma of bone. *Hum Pathol* 1980; 11: 581–3.
13. Young MPA, Freemont AJ. Primary leiomyosarcoma of bone. *Histopathology* 1991; 19: 257–62.
14. Wang TY, Erlandson RA, Marcove RC, Huvos AG. Primary leiomyosarcoma of bone. *Arch Pathol Lab Med* 1980; 104: 100–4.
15. WirbelRJ, Verelst S, Hanselmann R, Remberger K, Kubale R, Mutschler WE. Primary leiomyosarcoma of bone: clinicopathologic, immunohistochemical and molecular biologic aspects. *Ann Surg Oncol* 1998; 5: 635–41.
16. Torres FX, Kyriakos M. Bone infarct associated osteosarcoma. *Cancer* 1992; 70: 2418–30.
17. Pins MR, Mankin HJ, Xavier RJ, Rosenthal DI, Dickersin GR, Rosenberg AE. Malignant epithelioid hemangioendothelioma of the tibia associated with a bone infarct in a patient who had Gaucher disease. A case report. *J Bone Joint Surg* 1995; 77A: 777–81.
18. Abdelwahab IF, Hermann G, Kenan S, Klein MJ, Lewis MM. Case report 794. *Skeletal Radiol* 1993; 22: 379–81.
19. Abdelwahab IF, Klein MJ, Hermann G, Springfield D. Angiosarcomas associated with bone infarcts. *Skeletal Radiol* 1998; 27: 546–51.
20. Bullough PG, Kambolis CP, Marcove RC, Jaffe HL. Bone infarctions not associated with Caisson Disease. *J Bone Joint Surg* 1965; 47A: 477–91.
